# Penetration of SARS-CoV-2 Alpha, Delta, and Omicron variants in the United States

**DOI:** 10.1017/S0950268825100290

**Published:** 2025-08-04

**Authors:** Hosoon Choi, Munok Hwang, John D. Coppin, Piyali Chatterjee, Thanuri Navarathna, Emma Brackens, Lynn Mayo, Brandon Corona, Taylor Yakubik, Collin Telchik, Chetan Jinadatha

**Affiliations:** 1 Department of Research, Central Texas Veterans Health Care System, Temple, TX, USA; 2 Department of Medicine, Baylor Scott & White Medical Center – Temple, Temple, TX, USA; 3 College of Medicine, Texas A&M University, Bryan, TX, USA

**Keywords:** COVID-19, VOC, Variant of Concern, Penetration, Spread

## Abstract

The Alpha, Delta, and Omicron variants of the SARS-CoV-2 virus have been deemed as variants of concern (VOCs) by the WHO due to their increased transmissibility, severity of illness, and resilience against treatments. Geographically tracking the spread of these variants can help us implement effective control measures. RNA from 8,154 SARS-CoV-2 positive nasal swab samples from a Central Texas hospital collected between March 2020 and April 2023 were sequenced in Temple, TX. Global and U.S. sequencing metadata was obtained from the GISAID database on 3 April 2023. Using sequencing metadata, the growth rate of Alpha, Delta, and the first subvariant of Omicron (BA.1) were obtained as 0.27, 0.3, and 1.08 each. The average time in days to penetrate the US for Alpha, Delta, and Omicron were 269.2, 326.2, and 27.3 days, respectively. Viral sequencing data can be a useful tool to examine the spread of viruses. Each emerging SARS-CoV-2 variant penetrated cities more rapidly as the pandemic progressed. With a high logarithmic growth rate, the Omicron variant penetrated the US more rapidly as the pandemic progressed.

The COVID-19 pandemic initially began in December 2019 after a cluster of patients presented with an atypical pneumonia-like illness of unknown aetiology in China’s Hubei Province. Only 1 month after this novel coronavirus appeared in China, other countries reported laboratory-confirmed cases of SARS-CoV-2. By 19 January 2020, there were 282 confirmed cases in four countries: China, Thailand, Japan, and South Korea. Efforts to limit the spread of SARS-CoV-2 were quickly employed, including the screening of travellers; however, the first case in the United States was confirmed on 20 January 2020. What would later be declared by the WHO as a global pandemic – COVID-19 spread throughout the world with a total of 775,754,322 confirmed cases and 7,053,902 deaths worldwide as of 7 July 2024, according to the WHO.

While the mutation rate of SARS-CoV-2 was estimated at 1.3 × 10^−6^ ± 0.2 × 10^−6^ substitutions per base per infection cycle, it evolved rapidly and produced numerous variants. Waves of SARS-CoV-2 infection have been generated by emerging variants. The consecutive success of variants was mainly due to enhanced infectivity and the ability to evade immunity. The World Health Organisation (WHO) identified specific virus strains as variants of concern (VOC) based on their increased transmissibility, which causes more severe disease, and their ability to reduce the effectiveness of vaccines and treatments. As of December 2021, WHO identified five SARS-CoV-2 VOCs: Alpha, Beta, Gamma, Delta, and Omicron. While the temporal growth of each variant has been examined by estimating the effective and basic reproduction number (*R_e_* and *R*_0_) of each variant, studies about the expansion of infections have received less attention. However, the study of the geographical spread of infection waves can provide valuable information for effective control measures [1].

In this study, the weekly sequence data of the Alpha, Delta, and Omicron VOCs from Temple, TX from March 2020 to April 2023 were examined to obtain the growth rate of 3 major VOCs. The SARS-CoV-2 sequence database was utilized to investigate the penetration of COVID-19 across the United States.

Tracking the introduction and spread of variants helps monitor the dynamics of the COVID-19 pandemic, which can help assess the impact of variants on disease severity, transmission rates, and the burden on the local healthcare systems. Identifying the timing of variant introductions in local areas also allows for a more targeted public health response, such as adjusting testing strategies, implementing quarantine measures, enhancing contact tracing efforts, and evaluating treatment strategies. Studying COVID-19 transmission, even after the pandemic, is crucial for preventing future outbreaks of new variants or other respiratory viruses. Additionally, continued research helps inform public health policies, improve treatment options, and prepare for potential future pandemics [2].

Nasal swab samples were collected from inpatient wards, outpatient clinics, routine screenings, and employees (including some people who may be veterans) of Central Texas Veterans Affairs hospital in Temple, TX, as part of the COVID-19 response. All SARS-CoV-2 positive samples identified by quantitative PCR (qPCR) were subject to whole genome sequencing regardless of their PCR cycle threshold (Ct) value. SARS-CoV-2 RNA was extracted from swab samples using the QIAmp Viral RNA kit (Qiagen), and the library for sequencing was prepped using the COVIDseq test kit (Illumina) or the Swift Normalase kit (IDT). A sequencing run was performed using the Nextseq 500/550 mid output kit with paired-end reads of 149 cycles on a Nextseq 550 system. FASTQ files obtained after the sequencing run were reference mapped with a SARS-CoV-2 reference genome (NC_045512), and analysis for the samples’ lineage and mutation was performed with the DRAGEN COVID Lineage app within BaseSpace (Illumina). For some lower coverage samples, FASTQ files that were obtained using both the COVIDseq test kit and the Swift Normalase kit were combined [3].

The number of weekly world and U.S. SARS-CoV-2 sequence metadata for the Alpha, Delta, and Omicron VOCs were obtained from the GISAID hCoV-19 database (https://gisaid.org/) on 3 April 2023. The GISAID hCoV-19 database encompasses 215 countries from 6 continents. All VOCs included their sub-lineages as well. For the analysis of the first detection of VOCs, the cities were chosen to represent various U.S. regions. Since the detection date for each variant is based on the collection date of a sample, only GISAID data that included complete collection date and location information was utilized. Regions with limited information in the GISAID database could not be included in the analysis.

The logistic growth model was applied to the cumulative sum of cases each week to obtain growth parameter *k* (Alpha, Delta, Omicron BA.1) [4].

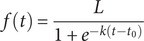

Where *t* is the time, *t_0_* is the time at the function’s midpoint, *k* is the logistic growth rate of infection, and *L* is the maximum cumulative sum of cases of the variant. The Bayesian models were programmed and fit in Stan, using rstan version 2.32.6, and parameter estimates are reported as means and central 95% intervals of the Markov Chain Monte Carlo samples. There are multiple waves of Omicron due to subvariants of Omicron. The first wave of Omicron by BA.1 was chosen for the analysis.

The time for each variant to reach each city was referred to as the days of penetration (*DP*). 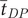 , the average time in days to penetrate all 21 U. S. cities, was calculated using the time-weighted mean [1].

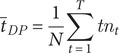

The equation for 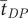 is shown above, where the first day in which the variant was detected in GISAID is *t* = 0 and subsequent days are *t* = 1, 2, …, *T*, where *T* is the number of days for each variant to penetrate all the U.S. cities examined. *N* is the number of U.S. cities examined (*N* = 21), and *n_t_* is the number of cities whose penetration occurred at day *t.*

The transmission pattern of SARS-CoV-2 was examined based on the sequence metadata for weekly Alpha, Delta, and Omicron VOCs uploaded to GISAID for comparison among the world, the United States, and the local Temple area. All positive samples from patients at the local hospital collected since the beginning of the pandemic were sequenced, including samples with high Ct value. Out of the 8,154 local samples collected between March 2020 and April 2023, 6,531 samples had higher sequence coverage to identify the lineages of SARS-CoV-2. The total number of samples, including all lineages from the United States in that period, was 4,628,399. The emergence patterns of VOCs were similar among the three regions of examination (Figure 1a). In the local Temple area, the Alpha VOC was detected in January 2021 and peaked around late March and early April 2021. However, the number of Alpha variants was low and occupied only 0.87% (57/6,531) of all the samples collected in the local Temple area, compared to 5.4% (250,049/4,628,399) of the samples from the United States. The Delta VOC wave started in March 2020 in the world and in the United States and reached its peak around July to August 2021. The Delta VOC was detected in late April 2021 in the local Temple area, and cases elevated around August to September 2021. The number of Delta variants was higher than that of the Alpha variant and occupied around 12.4% of all samples collected in the local Temple area. Delta variants consisted of 32.7% of all U.S. samples. The Omicron VOC was first detected in November 2021 and reached its peak in January 2022 in the United States and the world. For the local Temple area, the Omicron VOC was first detected in December 2021 and peaked in January 2022. The number of Omicron variants was much higher compared to the previous two VOCs and comprised around 67% of all samples collected in the local Temple area and 49.9% of all the U.S. samples. The emergence of the Omicron variant was sudden, while the Alpha and Delta variants were more gradual.

**Figure 1.**
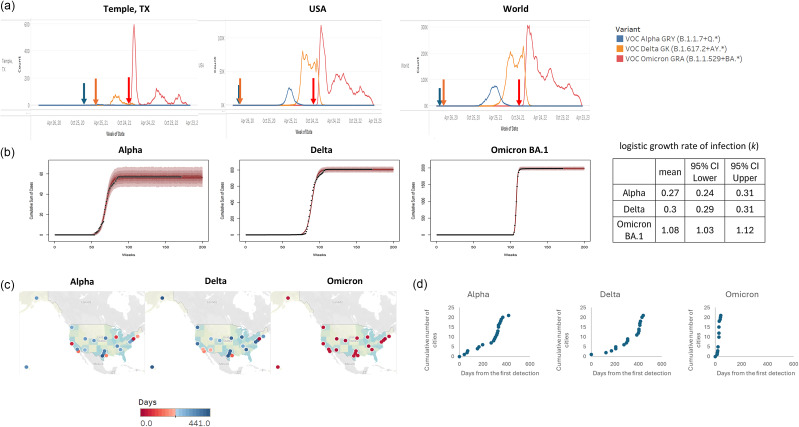
The changes in prevalence of Alpha, Delta, and Omicron VOCs in the local catchment area and their spread in the US. (a) The number of VOCs in the local Temple, TX region. The number is compounded weekly from March 15, 2020, to April 2, 2023. Arrows indicate first the detection date of each variant. (b) Modelling results for time-series data of VOCs. (c) Penetration of VOCs to major cities in the United States. Days were calculated using the difference between the first detection date of each variant in the world and the first appearance of each variant in each city. The red gradients indicate early emergence while blue gradients indicate late arrival. (d) The graphs plot the cumulative number of cities in which each of the three variants was first detected.

To obtain epigenetic characteristics of VOCs, we applied a logistic growth function to analyse time-series SARS-CoV-2 sequencing data. The growth rate of Alpha, Delta, and Omicron BA.1 in Temple area were 0.27 (0.24, 0.31), 0.30 (0.29, 0.31), and 1.08 (1.03, 1.12) each (Figure 1b).

The SARS-CoV-2 transmission over the United States was examined by exploring the first detection date of each VOC for 21 cities across the United States for which sequence collection data available in the GISAID database.

Overall, it took longer for the emergence of the Alpha and Delta variants to occur in all major U.S. cities than it took for the emergence of Omicron, which happened much faster. The spatial expansion of each SARS-CoV-2 variant is represented with time-weighted mean days of penetration, with 269.2 days and 326.2 days for the Alpha and Delta variants, respectively. The mean days of penetration for the Omicron variant was 27.3 days. Table 1 shows the WHO designation dates for the Alpha, Delta, and Omicron VOCs and the first detection date for each VOC in the world. First detection dates and days to penetration for each VOC were also listed for each selected U.S. city. The days to penetration were calculated as the difference between the first detection date of each variant in the world and the date that each variant appeared in each U.S. city. The first three cities showing the emergence of the Alpha variant were marked with red, orange, and yellow, respectively, in Table 1 to emphasize the differences in transmission trends among the VOCs. The Texas cities were highlighted in green in Table 1 to demonstrate that even in well-connected and relatively closely located cities, the penetration of each VOC is not closely related, which may reflect the heterogenetic nature of COVID-19 transmission. The emergence patterns were different for the Alpha and Omicron VOCs. The first detection of the Alpha and Delta variants in the United States was in New York City, NY, while the first detection of Omicron was in Nashville, TN. Figure 1c geographically depicts the days until emergence of each VOC. The darker shades of red in the gradient indicate a smaller number of days until penetration, while the darker shades of blue indicate a larger number of days until penetration. Among the four Texas cities, only Houston showed a smaller number of days up until penetration for the Alpha and Delta VOCs, while Dallas, San Antonio, and Temple showed a larger number of days until penetration for the same VOCs. The graphs in Figure 1d are spatial growth curves formed by plot the cumulative count of cities by days of earliest detection of the Alpha, Delta, and Omicron variants. In the local Temple area, it took 343, 404, and 42 days for the emergence of the Alpha, Delta, and Omicron variants, respectively.

**Table 1.** Penetration of the Alpha, Delta, and Omicron variants in major cities in the United States

VOC Alpha GRY (B.1.1.7 + Q.^a^)			VOC Delta GK (B.1.617.2 + AY.^a^)			VOC Omicron GRA (B.1.1.529 + BA.^a^)		
WHO VOC date: 12/18/2020			WHO VOC date: 5/11/2021^b^			WHO VOC date: 11/26/2021^b^		
Region	First detection date^c^	Days to first penetration	Region	First detection date^c^	Days to first penetration	Region	First detection date^c^	Days to first penetration
World	2/7/2020	0	World	3/12/2020^d^	0	World	11/1/2021	0
New York, NY	3/19/2020	41	New York, NY	3/12/2020	0	Nashville, TN	11/13/2021	12
San Francisco, CA	4/12/2020	65	Houston, TX	7/14/2020	124	Phoenix, AZ	11/17/2021	16
Chicago, IL	4/17/2020	70	San Diego, CA	8/31/2020	172	Salt Lake City, UT	11/18/2021	17
San Diego, CA	7/8/2020	152	Phoenix, AZ	10/5/2020	207	Denver, CO	11/22/2021	21
Houston, TX	7/21/2020	165	Los Angeles, CA	10/6/2020	208	New York, NY	11/22/2021	21
Boston, MA	8/21/2020	196	Salt Lake City, UT	12/16/2020	279	Los Angeles, CA	11/27/2021	26
Phoenix, AZ	11/4/2020	271	Denver, CO	1/4/2021	298	Honolulu, HI	11/27/2021	26
Seattle, WA	11/23/2020	290	Nashville, TN	1/14/2021	308	Boston, MA	11/27/2021	26
Washington, DC	12/1/2020	298	Boston, MA	1/16/2021	310	San Antonio, TX	11/27/2021	26
Atlanta, GA	12/10/2020	307	San Francisco, CA	4/3/2021	387	Seattle, WA	11/27/2021	26
Anchorage, AK	12/20/2020	317	Seattle, WA	4/3/2021	387	San Francisco, CA	11/28/2021	27
Denver, CO	12/24/2020	321	Philadelphia, PA	4/17/2021	401	Philadelphia, PA	11/28/2021	27
Salt Lake City, UT	12/31/2020	328	Temple, TX	4/20/2021	404	Anchorage, AK	11/29/2021	28
Los Angeles, CA	1/4/2021	332	Atlanta, GA	4/22/2021	406	Washington, DC	11/29/2021	28
Philadelphia, PA	1/4/2021	332	Washington, DC	4/28/2021	412	Omaha, NE	11/29/2021	28
Nashville, TN	1/5/2021	333	Chicago, IL	4/28/2021	412	Atlanta, GA	11/30/2021	29
Temple, TX	1/15/2021	343	Dallas, TX	4/30/2021	414	Chicago, IL	11/30/2021	29
Honolulu, HI	1/21/2021	349	Omaha, NE	5/3/2021	417	Houston, TX	11/30/2021	29
Dallas, TX	1/27/2021	355	Anchorage, AK	5/18/2021	432	San Diego, CA	12/7/2021	36
Omaha, NE	2/10/2021	369	Honolulu, HI	5/18/2021	432	Dallas, TX	12/9/2021	38
San Antonio, TX	4/1/2021	419	San Antonio, TX	5/27/2021	441	Temple, TX	12/13/2021	42
Average number of days to penetrate all 21 U.S. cities ( 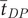 )		269.2			326.2			27.3

a The VOCs include their sub-lineages.

b Prior to VOC designation, VOI (Variant of Interest) designation was on April 4, 2021 for Delta and VUM (Variant under Monitor) designation was on November 24, 2021 for Omicron.

c Detection date is based on the sample collection date.

d The initial detection date of Delta has changed as sequence information is updated. The first city of Alpha penetration, New York is marked in red. The second and the third cities, San Francisco and Chicago, are marked in orange and yellow, respectively. Green indicates cities in Texas.

Viral mutations are crucial for the survival and propagation of a virus. Continued surveillance on both a global and local level is imperative, as the Alpha, Delta, and Omicron variants each contained mutations that conferred either increased transmissibility, higher severity of the disease when infected, increased risk of death, decreased neutralizing activity by vaccine-induced immune response, or some combination of these features. The N501Y substitution in the S protein allowed for more efficient transmission. Additionally, mutations present in the Delta variant conferred higher pathogenicity and higher viral loads.

Phylogenetic analyses suggest that the Alpha variant, first discovered in September 2020 in the United Kingdom and detected in multiple countries around the world within months, originated and spread globally from the United Kingdom. Spread of the virus was likely facilitated by global connectivity and high levels of human mobility. Further genetic analysis indicates that the Alpha variant was first detected in February 2020, reaching the United States in March 2020, and subsequently the local Temple area 343 days later on 15 January 2021. The Delta VOC similarly reached the local Temple area on 20 April 2021, 404 days after its initial detection date in the United States. Interestingly, the data show that the Omicron variant appeared in Temple only 42 days after the first detection date in the United States. This penetration to the local Temple area and across the United States was much faster than the time to penetration observed for the Alpha or Delta variants. This may be due to several factors. First, Omicron has more mutations compared to prior VOCs. Omicron’s mutations may confer increased transmissibility. Second, public health restrictions that were implemented throughout the pandemic to mitigate the spread had loosened during Omicron compared to earlier VOCs.

The Omicron variant is the most heavily mutated SARS-CoV-2 variant with >97% of mutations present in the coding region and several mutations within the spike protein [5]. One study performed a mutational analysis noting the average number of mutations per Omicron genome was 60.5, whereas the average number of mutations for Delta and Alpha genomes was 39 and 30.7, respectively, although these were calculated using limited genomes [5]. Some of the mutations present in the Omicron variant were also found in previously described VOCs that conferred the ability of the virus to escape neutralization by vaccines and convalescent serum. Furthermore, the Omicron variant has additional novel mutations along with those identified in prior VOCs. The rapid spread of the Omicron variant, which the data support as the variant was detected in Central Texas a mere 42 days after initial detection in the United States, suggests increased transmissibility. One study found that Omicron had a 3.31-fold higher reproduction number than that of Delta in South Africa. The transmissibility of Omicron was considerably higher than previous VOCs. Omicron reached the outbreak threshold 5 to 10 days after initial identification, whereas other variants reached the outbreak threshold between 14 and 35 days after first detection [6]. This data supports the observation that Omicron penetrated much faster than prior variants and reached a higher total number of cases than other variants in the local community. Despite the rapid transmission and increase in cases, multiple studies have shown decreased pathogenicity of the Omicron variant [6]. One study in a hamster model showed that the Delta variant was more pronounced in bronchial epithelium and alveolar spaces, whereas Omicron was only sporadically found in those spaces and was less efficiently transmitted between epithelial cells [6]. The impacts of the Delta and Omicron variants in global and local communities have been pronounced, possibly owing to the increased pathogenicity observed with the Delta variant and the increased transmissibility noted with the Omicron variant in the local community. While standard compartmental models of infectious diseases show infectious populations are diminished after a single wave, actual SARS-CoV-2 infections have several waves partially due to the formation of diverse variants that are antigenically distinct ‘quasispecies’ with increased viral fitness achieved by avoiding human immune response generated by both vaccination and prior infection [7]. As a result, repetitive waves of infections moved over each local area. Multiple waves of infections should be anticipated in the management of the next pandemic.

In such a long and extensive pandemic, it has been necessary to adjust strategies such as lockdowns, isolations, and quarantines. These non-pharmaceutical interventions have been exercised during the multiple pandemic waves to mitigate the spread of COVID-19. They have often been applied to large and diverse territories. However, the spread of SARS-CoV-2 infections is highly heterogeneous [8]. There are geographical differences in arrival time, incidence, and mortality rates. In large territories, the effectiveness of controlling measures was highly varied across the constituting regions [9]. Moreover, administration of vaccines against SARS-COV-2, which were administered in different countries at varying times as a prevention strategy, may also bear an impact on reducing transmission. However, newly emerging variants that contain mutations that mediate escape from vaccine-induced immunity, make the effect of vaccines on the prevention of transmission less pronounced. Accurate and timely estimation of the speed of transmission of the multiple waves of a pandemic is crucial for predicting regional spread of infection and evaluating and adjusting control measures. Clear understanding of the spatiotemporal spread of COVID-19 should be highly considered before major decision making, planning, and community action.

In order to provide information on the spatiotemporal spread of multiple variants, a national-level genomic surveillance network should be established in preparation for future pandemics [10]. A genomic surveillance network would enable us to track infection transmission, identify viral mutations and variants, predict disease development trends, and provide essential knowledge of epidemic dynamics.

Several possible limitations to this study exist. Data used in this study was based on sequence data from the local sequencing facility and from GISAID and was not representative of all infection cases since sequence data were not collected through random sampling. The lineage of SARS-CoV-2 can only be obtained if samples are sequenced with adequate sequencing coverage. Areas without sequencing facilities or the ability to send samples for sequencing may have been excluded, skewing the data as well as early emergence dates. In addition, not all available sequencing data may have been uploaded to public databases. As such, even the most popular public SARS-CoV-2 database, like GISAID, cannot represent all COVID-19 patients and variants. Another limitation of this study is that the local data was collected from a Veterans Affairs hospital, which reflects the veteran population in the area and may not reflect the trends of the entire local civilian population. The initial detection date also can change as more sequencing information is uploaded to the GISAID database and analysed, which may mean that initial detection dates may change as follow-up sequencing efforts continue.

In summary, using SARS-CoV-2 sequencing data entry as a proxy for infection count, the spread of SARS-CoV-2 Alpha, Delta, and Omicron VOCs was examined. As growth rates calculated from local sequencing data increased to 0.27 (0.24, 0.31), 0.30 (0.29, 0.31), and 1.08 (1.03, 1.12) for Alpha, Delta, and Omicron VOCs, the penetration of each variant became faster as the pandemic progressed, and the Omicron VOC was widespread in all major U.S. cities within a month of its known emergence.

## Data Availability

The datasets used and/or analysed during the current study are available from the corresponding author on reasonable request.
